# Similarities and Differences between Silver Ions and Silver in Nanoforms as Antibacterial Agents

**DOI:** 10.3390/ijms19020444

**Published:** 2018-02-02

**Authors:** Anna Kędziora, Mateusz Speruda, Eva Krzyżewska, Jacek Rybka, Anna Łukowiak, Gabriela Bugla-Płoskońska

**Affiliations:** 1 Department of Microbiology, Institute of Genetics and Microbiology, University of Wrocław, 51-148 Wrocław, Poland; mateusz.speruda@uwr.edu.pl; 2 Department of Immunology of Infectious Diseases, Hirszfeld Institute of Immunology and Experimental Therapy, Polish Academy of Sciences, 53-114 Wrocław, Poland; eva.krzyzewska@iitd.pan.wroc.pl (E.K.); rybka@iitd.pan.wroc.pl (J.R.); 3 Institute of Low Temperature and Structure Research, Polish Academy of Sciences, 50-422 Wrocław, Poland; a.lukowiak@int.pan.wroc.pl

**Keywords:** silver ions, silver nanoparticles, silver nanocompounds, nanotechnology, mode of action, resistance of bacteria

## Abstract

Silver is considered as antibacterial agent with well-known mode of action and bacterial resistance against it is well described. The development of nanotechnology provided different methods for the modification of the chemical and physical structure of silver, which may increase its antibacterial potential. The physico-chemical properties of silver nanoparticles and their interaction with living cells differs substantially from those of silver ions. Moreover, the variety of the forms and characteristics of various silver nanoparticles are also responsible for differences in their antibacterial mode of action and probably bacterial mechanism of resistance. The paper discusses in details the aforementioned aspects of silver activity.

## 1. Introduction

Silver ions as antibacterial agents have been known for ages. A detailed history of silver usage was well documented in the literature [1,2]. Silver ions from dissolved silver nitrate (lapis, AgNO_3_) and silver sulfadiazine are agents with proved efficacy against Gram-positive and Gram-negative bacteria [3,4,5,6]. 

The dynamic development of nanotechnology in recent years has provided possibilities for fabricating various forms of silver nanoparticles (AgNPs) [7]. Their most important feature is the highly developed surface area of small-size particles, which allows to increase the antimicrobial efficacy and bioavailability of materials used in the biology and biomedical sector [8]. We can observe a “nanotechnology race” of nanoproducts applications [7] in the biomedical sector too. In 2017 Sheng et al. [9] reviewed that over 1000 articles concerned the effects of nanoparticles on bacteria and over 90% of them were published after 2008. At present, more and more publications concerning the synthesis and antibacterial activity of silver nanoforms are being published [10,11,12,13,14,15,16,17,18,19,20,21,22,23,24,25,26,27,28,29,30,31,32].

Therefore, the main goal of this manuscript is to emphasize the variety of silver nanomaterials, their diversity in physico-chemical properties and finally, the high possibility for different interactions with cells—especially with bacterial pathogens. We compared the mode of action of silver ions and silver nanocomposites against bacteria (Gram-positive and Gram-negative bacterial cells), analysed the limitation of usage of silver materials considering the bacterial resistance to them, determined the gap of knowledge and showed future perspectives. Increasing amount of silver in the environment, having its source in human activities, especially nanotechnology products, cause air, water and earth pollution. We would like to emphasize the substantial differences between various forms of silver nanoparticles which cannot be considered as identical materials. As in the case of other biocides the border between benefits and toxic effect is very narrow [33]. 

## 2. Properties of Silver Materials vs. Antibacterial Activity

Silver ions are relatively reactive. The binding of silver ions in the form of insoluble precipitates (AgCl), or during the interactions with proteins (e.g., albumin), causes a significant decrease of its antibacterial efficacy, which is very important in the case of Ag^+^ bioapplications. 

AgNPs can exist in the form of metallic silver with atoms strongly connected with each other [34]. The typical AgNPs are usually of a few to a dozen nm in diameter [35] and can take different shapes (spherical, irregular or planar) [9,20,24,25,26,27,28,36,37,38] that depend on the methods of production. One of the most popular “bottom up” eco-friendly method is biological synthesis, using living cells of bacteria, fungi and plants to obtain silver nanoparticles [9,15,16,17,19,24,27,37,38].

It is worth to underline that AgNPs never perform alone due to their tendency to aggregate as a result of the interactions between silver atoms. To prevent aggregation organic (e.g., citric acid) or inorganic carriers (stabilizers, such as silica, graphene or titanium dioxide) are used. From the point of view of nanotechnology, the unaffected physico-chemical properties of AgNPs (especially large surface area to volume ratio) are crucial to maintain their antibacterial efficacy. Variety of synthesis methods (including chemicals and reaction parameters) determine the variety of physico-chemical properties of nanomaterials and their biological activity. In Table 1 the influence of important chemical and physical properties on that activity is shown.

## 3. Mode of Antibacterial Action of Silver (MoA)

Li et al. [39] proved that silver ions have similar mode of action to silver nanoparticles but stronger antibacterial activity than AgNPs.

The antibacterial activity of silver ions (Ag^+^) is directly proportional to the environmental concentration of silver ions. Due to the oligodynamic effect, silver shows high antibacterial efficacy even in low concentrations. Jung et al. [40] compared the antibacterial activity of silver ions obtained in various ways and showed that silver ions produced in an electrolytic way are better antibacterial agents than those obtained by dissolving the silver compounds. 

**Figure 1:**
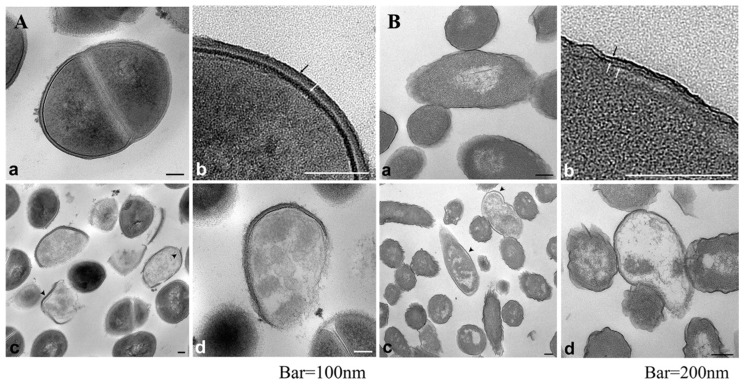
Internal morphology of *S. aureus* (**A**) and *E. coli* (**B**) observed via TEM (**a**,**b**) untreated bacteria, (**c**,**d**) bacteria treated with Ag^+^ (0.2 ppm) during 2 h. Black and white arrows indicate peptidoglycan and cytoplasmic membrane, respectively (**A**) and outer membrane, peptidoglycan and cytoplasmic membrane (**B**). Arrowhead indicate separation of the cell membrane from the cell wall. Reprinted from [40] with American Society for Microbiology Publishing Group permission.

The antibacterial mode of action of silver ions is connected with: (i) interaction with the bacterial cell envelope (destabilization of the membrane—loss of K^+^ ions and decrease of ATP level, bonded with phospholipids), (ii) interaction with molecules inside the cell (e.g., nucleic acids and enzymes), (iii) the production of reactive oxygen species (ROS) [41]. The interaction of silver ions with bacterial inner membrane is one of the most important mechanisms of Ag^+^ toxicity [42]. Jung et al. [40] proved that the accumulation of Ag^+^ in the bacterial cell envelope is followed by the separation of the cytoplasmic membrane (CM) from the cell wall in both Gram-positive and Gram-negative bacteria [40,43]. Sütterlin et al. [41] showed that a minimal bactericidal concentration (MBC) of Ag^+^ for Gram-positive bacteria was more than 32 times higher than the MBC values for the Gram-negative bacterial cells. According to reference [40], carboxyl groups (–COOH) in glutamic acid and phosphate groups in teichoic acid are mostly responsible for binding of silver ions. On the other hand, Randall et al. [44] suggested that the damage caused by Ag^+^ in the inner membrane (IM) is one of the most important mechanisms in staphylococci. It has been proved that silver ions enter bacteria cells within 30 min of exposition and bind to cytoplasm components, proteins and nucleic acids [40,45]. As it can be seen from the TEM (transmission electron microscopy) pictures, shown in Figure 1, both types of bacterial cells (Gram-positive and Gram-negative) treated with Ag^+^ were lysed and the leakage of cytoplasm could be observed in all cases. Jung et al. [40] suggested that silver ions induce an ”active but nonculturable” state (ABNC) in bacteria cells. Stress induced by Ag^+^ caused that bacteria maintained the metabolism and physiology but stopped the growth, therefore the number of viable cells decreased in the performed in vitro tests. 

One of the differences between the mode of Ag^+^ action against Gram-positive and Gram-negative bacteria regards the way of silver uptake into the cell. Silver ions enter Gram-negative cells via major outer membrane proteins (OMPs), especially OmpF (and its homolog OmpC) [21,43], which is a 39 kDa transmembrane protein with trimeric β-barrel structure. Each monomer of OmpF is built by sixteen transmembrane, antiparallel β-strands assembled with each other via hydrogen bonds. Those strands form a stable β-sheet which afterwards folds into a cylindrical tube with a channel function. Besides porin and ion transporter activity, OmpF is involved in the transport of other small molecules (e.g., drugs) across the bacterial outer membrane (OM) [46,47]. The importance of the OmpF/OmpC role in the mechanism of resistance to silver has been discussed repeatedly in a few published papers [43,48,49,50]. Sometimes the results of the conducted experiments were quite different. Radzig et al. [48] claimed that *E. coli* lacking OmpF (or OmpC) in the OM was 4–8 times more resistant to Ag^+^ or AgNPs than *E. coli* which possessed those proteins. In another study, Randall et al. [43] proved that prolonged exposure to silver ions caused missense mutations in the *cusS* and *ompR* gene. The latter resulted in the loss of function of OmpR protein (which is a transcription factor of OmpF and OmpC) and, finally, in the lack of OmpF/C proteins in the OM. *E. coli* BW25113 without the mentioned OMPs is characterized by a low permeability of the OM and a high level of resistance to Ag^+^. Those features were observed only in the situation when both proteins were not present in the OM [43]. Yen et al. [49] stand in opposition to the results shown above. In their research, regardless of the presence or absence of OmpF/OmpC in the bacterial OM, they observed no changes in bacterial sensitivity to silver ions. Li et al. [50] tested the antibacterial activity of silver-coated carbon nanotubes on *Salmonella* Typhimurium and observed reduced expression of the *ompF* gene after exposure to these nanoparticles. 

Another molecular mechanism of antibacterial toxicity of silver ions is connected with their interaction with structural and functional proteins, especially those with thiol groups (–SH) [42,45,51]. Inhibition of the main respiratory chain proteins (e.g., cytochrome b) causes an increase of ROS inside the cell, what contributes to the death of bacteria. Exposure to silver results in the increase of the level of intracellular reactive oxygen species, what leads to oxidative stress, protein damage, DNA strand breakage, and, consequently, cell death [45]. One of the major targets inside the cell is the S2 protein, localized in small subunits of the bacterial ribosome. The binding of silver ions to ribosomal proteins results in the denaturation of the ribosome native structure and inhibition of protein biosynthesis [45]. Moreover, it has been proved that silver ions interact with nucleic acids forming bonds with pyrimidine bases. In the consequence, DNA condenses and replication is inhibited [52].

**Figure 2:**
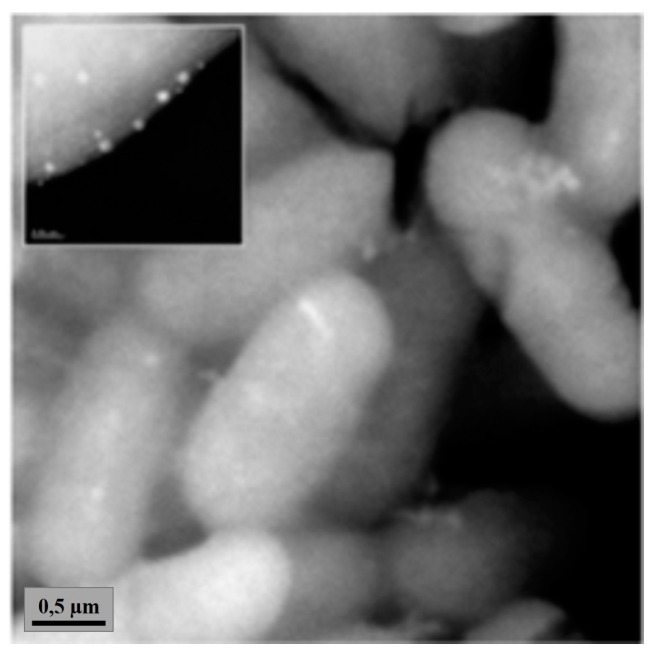
Accumulation of silver nanoparticles in *P. aeruginosa* cells (silver nanoparticle concentration 75 µg/mL, silver size: 10 nm). Reprinted from [23] with Copyright Clearance Center permission.

The antibacterial mode of action of silver nanoparticles remains still unclear and is the subject of discussion. A lot of science reports suggests that the mechanism of toxicity of AgNPs is similar to silver ions, due to the life cycle of silver nanoparticles and their transformation to silver ions [22,23,53,54]. Silver nanoparticles react with Gram-negative and Gram-positive bacteria cells in the following way: (i) with the cell envelope (e.g., membrane, peptidoglycan, Figure 2), (ii) with significant structure molecules (e.g., proteins, nucleic acids) and (iii) in biochemical pathways [20,21,23,35,55,56,57]. Shrivastava et al. [18] suggested that one of the possible antibacterial modes of silver nanoparticles action is the inhibition of signal transduction and growth (noted only in Gram-negative bacteria) by dephosphorylation of the peptide substrates on tyrosine residues.

One of the most important ways of silver antibacterial activity is the induction of ROS production. This effect in the case of silver ions was partially described in this chapter. AgNPs induce the higher concentration of hydrogen peroxide (H_2_O_2_), superoxide anion (O_2_^−^•) and hydroxyl radical (OH•) inside bacterial cell [52,58,59]. The detailed mechanism is still not well known but superoxide is predicted to be the major ROS in this process [52,60]. AgNPs disturb the function of the respiratory chain in the cell resulting in ROS generation. When the level of ROS exceeds the capacity of the cellular antioxidant defence system (for example, through depletion of glutathione, GSH, and protein-bound sulfhydryl groups and changes in the activity of various antioxidants), oxidative stress occurs, leading to different ways of inhibition of cell proliferation. Ramalingam et al. [58] in their research tested biosynthesized AgNPs (9.1 ± 1.6 nm). They proved that the minimal concentration of AgNPs required for the induction of the reactive oxygen species production stands at 1.35 µg/mL. Higher AgNPs levels result in a depletion of GSH—an antioxidant crucial in neutralization of the free-radical species. Park et al. [61] claimed that the superoxide anion was the main form of reactive oxygen that cause bacterial cell death. Furthermore, they proved that H_2_O_2_ was not produced in the bacterial cell after the exposure to Ag^+^. The mechanism of hydrogen peroxide generation refers to bacteria exposed to atmospheric oxygen and the antibacterial activity of silver nanoparticles against anaerobes (in the lack of oxygen) is very low. It is important that AgNPs can induce an additional, exogenous, ROS generation. As nanoparticles—such as TiO_2_ or ZnO—with large surface areas and highly reactive catalytic sites can produce ROS in the presence of UV light due to photocatalytic properties [62], also the photocatalytic ROS generation by silver forms cannot be excluded [63].

Lok et al. [54] pointed out the high antibacterial activity of AgNPs and claimed that the effective concentrations of AgNPs and Ag^+^ (under aerobic condition) were at nanomolar and micromolar levels, respectively. Rai et al. [56] reviewed that silver nanoparticles may be also used against multidrug resistant (MDR) bacteria, both Gram-positive (methicillin-resistant *S. aureus*—MRSA, *Streptococcus pyogenes*) and Gram-negative (*E. coli*, *K. pneumoniae*, *P. aeruginosa*). 

Mandal et al. [64] proved the charge-dependent mechanism of AgNPs efficacy. They noticed the Zeta potential of *Enterococcus faecalis*, *Proteus vulgaris* and AgNPs on the level: −15, −26, −32.2 mV, respectively, which testify the charge cells and particles and indicated that Gram-positive *E. faecalis* cell accumulate more AgNPs than Gram-negative *P. vulgaris.* Sondi et al. [22] claimed that also negatively charged silver (silver nanocomposites), in comparison to positively charged silver ions, shows high antibacterial efficacy as well. 

The lifecycle of nanoparticles and nanocomposites has an important influence on the antimicrobial mode of action and efficacy and depends on the environmental conditions. Gitipour et al. [65] observed that spherical AgNPs (3–5 nm), used as a disinfectant in mouthwash, undergo transformation (aggregation) after usage. The inner diameter of AgNPs increased to 50–200 nm and chemical transformation of AgNPs to AgCl after usage was observed, what can prove the ionization of metallic silver from nanoparticles to silver ions. Biotransformation of silver nanoparticles depending on environmental condition is often observed. Mokhena et al. [26] observed that size and shape of nanoparticles changed after heating at 90 °C. After 3 h of heating, the size of AgNPs increased from 28 to 30 nm and spherical particles became irregular: their shape changed from spherical to rod-like. Heating for 48 h resulted in a mixture of rod-like (76–121 nm) with less abundant spherical (28–50 nm) nanoparticles. Raman et al. [27] indicated that during the eco-friendly production of silver nanoparticles their size and shape were pH and temperature dependent. McGillicuddy et al. [66] reported that AgNPs released from consumer products to the environment during their lifecycle have varied properties, although their determination is difficult. 

The theory about antibacterial mode of action of AgNPs is also connected with the oxidation of AgNPs. It is more than likely that the surface of silver nanoparticles is oxidized [57]. The smaller the size of fabricated particles, the higher oxide content due to larger surface area to volume ratio. The presence of oxide on the surface ensures high antibacterial activity of AgNPs, most probably due to the higher concentration of ROS [55] generated. Xiu et al. [67] showed that toxicity of AgNPs depends on the presence of O_2_ and is connected with silver ions release. They tested glycol-thiol-coated (PEG) AgNPs with different sizes. The oxidative dissolution of AgNPs was observed only under aerobic conditions and PEG coating did not secure nanoparticles from that phenomenon. In anaerobic conditions, tested PEG-AgNPs did not show the antibacterial activity upon *E. coli* K-12 due to nonoccurrence of dissolved silver ions. Xiu et al. [67] claimed that among other the size and shape or AgNPs coating has an influence on extent and duration of Ag^+^ release into solution. It was shown that the release of Ag^+^ ions from nanosilver in aqueous solutions corresponds to the mass leached or dissolved of one or two oxidized monolayers from its surface depending on nanosilver size. The antibacterial activity (against *E. coli*) of nanoparticles (size < 10 nm, where Ag_2_O layer was removed), was significantly lower than that of as-prepared particles with oxidized surface [68]. 

Rai et al. [56] and Durán et al. [69] reported that antibacterial efficacy depends on the size, shape, concentration and doses of used AgNPs. Morones et al. [23] observed the highest efficacy for AgNPs with a size below 10 nm. Pal et al. [20] showed the shape-dependent efficacy of AgNPs, which results from the different surface area between spherical and triangular AgNPs, with the latter form exhibiting much higher efficacy. Sheng et al. [9] reviewed in details the dose-response of bacteria to AgNPs and noticed high differences in the efficacy concentrations of different AgNPs and various cell response. 

Every nanocomposite has a different physical (size, shape, amount) and chemical (presence of other compounds, oxidation state) properties [9,21,28] (Table 1). Therefore, we speculate that they should be considered as separate forms, with different properties and different ways of bacterial toxicity. Thus, the mode of action of particular silver nanoparticles (nanoforms, nanocomposites) could be the result of the binding strength, type of target, time of interaction, oxidation level, etc. Moreover, the carrier’s compound can enhance the antibacterial activity due to changes of the physico-chemical properties as well as mechanical mode of action (for example, TiO_2_ in anatase form possess photoactivity and graphene-based structures might cause mechanical damages of the cell, overwrap bacteria or increase the surface area resulting in stronger interaction with cells [28,62]. When bioactivity of metal nanoparticles is considered, it is worth to mention that these materials can change their properties and toxicity depending on the time and conditions of storage. Both these parameters, as well as surface chemistry of AgNPs, influence the evolution of the nanoparticles’ properties over time. While stored, different processes may occur, such as oxidation, dissolution, agglomeration, capping agent degradation or attachment of Ag^+^ ions to container walls [70]. All the changes have a significant influence on the particles toxicity [54,70] indicating strong ”aging” effects. Therefore, contradictory toxicity results might be observed in the literature for identical AgNPs against the same bacteria.

**Figure 3:**
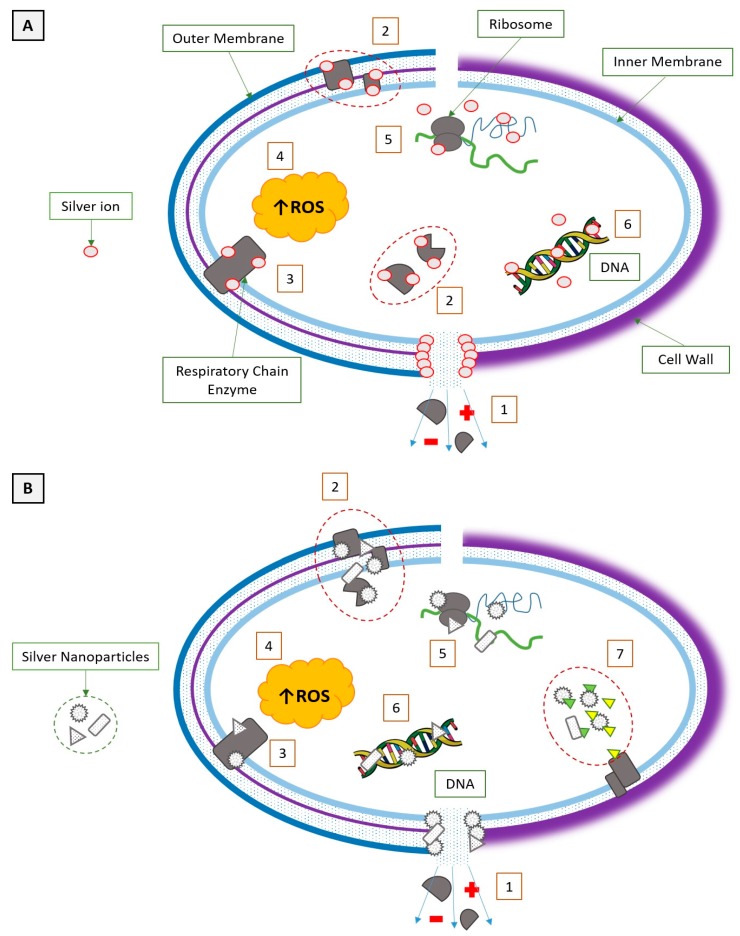
A comparison of the silver ions (**A**) and silver nanoparticles’ (**B**) mode of action to Gram-negative (**left**) and Gram-positive (**right**) bacteria. (1) Pore formation; metabolites and ions leakage (shown as plus and minus in the figure above) (2) Denaturation of structural and cytoplasmic proteins; enzymes inactivation. (3) Inactivation of respiratory chain enzymes. (4) Increase of intracellular reactive oxygen species (ROS) concentration. (5) Interaction with ribosome. (6) Interaction with nucleic acids. (7) Inhibition of signal transduction.

A comparison of the mode of action of silver ions and silver nanoparticles against Gram-negative and Gram-positive bacteria with short interaction description is presented in Figure 3.

## 4. Current Limitations and Future Prospects of Silver Materials Usage

**Figure 4:**
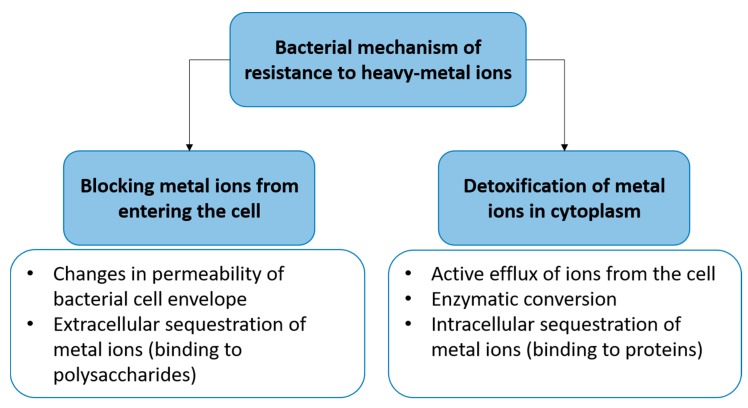
Diversity of bacterial mechanisms of resistance to heavy metals [71].

The general resistance of bacteria to heavy metals is presented in Figure 4. The mechanism of resistance to heavy metals is connected with the locking of uptake metal into the cell or the detoxification of the metals inside. The resistance of bacteria to silver may be divided into an endogenous and exogenous mechanism. The first one (endogenous) is connected with a loss of special proteins (OmpC/F) and an up-regulation of efflux mechanism (Cus system) as an effect of two point mutation after long-term exposition of bacteria (e.g., *E. coli*) to silver ions (AgNO_3_). The endogenous mechanism of bacterial resistance to silver ions was proven by Randall et al. [43]. They showed that silver provide selective pressure to enrich a population of silver resistant bacteria. After 6 days of exposure to subinhibitory concentrations of silver ions, the resistant strain of *E. coli* BW25113 could be selected. 

The exogenous way is associated with Sil proteins located in the cell membranes and responsible for efflux of silver ions out of the bacterial cell. The description of those mechanism is located below (Figure 4).

The chromosome-encoded mechanism of resistance to silver is strictly connected with the presence of efflux pumps within bacterial membranes [43]. Due to its functions, similarities in protein sequences, substrate specificity and subcellular location, all efflux pumps were classified into five superfamilies: ABC (ATP-binding cassette), MFS (major facilitator superfamily), RND (resistance-nodulation-division), MATE (multidrug and toxic compound extrusion) and SMR (small multidrug resistance) [72]. The pumps of each mentioned superfamily were already found in the cell of Gram-negative bacteria [50]. Transporters responsible for the extrusion of drugs, detergents, biocides, dyes and, importantly, heavy metals into extracellular space belong to the resistance-nodulation-division superfamily. The activity of those pumps depends on the proton-motive force [72,73]. Those transporters have the ability to capture toxic compounds from cytoplasm and the periplasmic space. RND pump consists of three subunits: substrate-binding transporter (located in the inner membrane), periplasmic membrane fusion protein (MFP) and outer membrane factor (OMF). The formed complex seems to be spanning both membranes [73,74]. RND transporters with high specificity to toxic cations belong to the HME-RND (heavy-metal efflux) subfamily, such as CusCFBA—one of few HME-RND pumps, responsible for bacterial resistance to copper and silver ions [75,76,77]. 

The operon *cusCFBA* of a bacterial chromosome encodes the system of active, extracellular transport of Ag(I) and Cu(I) which consists of CusA, CusB, CusC (proteins, subunits of RND efflux pump) and a periplasmic chaperone, CusF [75,78]. The transcription of genes encoding CusCFBA depends on the extracellular concentration of heavy-metal ions and the occurrence of operon *cusRS*. The operon gathers genes of CusR (response regulator) and CusS (histidine kinase) which regulate the expression of the efflux pump’s components after reaction with the stimulant [79,80]. Thus far, CusCBA is the only known chromosomal-encoded pump responsible for silver resistance [75,80].

The function and structure of each CusCFBA subunit and CusF is well known. The substrate-binding transporter, CusA, is located in the inner membrane. This homotrimer consists of 1047 amino acids and contains 12-transmembrane α-helices [75,76,80]. Its activity depends on proton-motive force. Heavy-metal ions bind to the three-methionine motif (M573, M623, M672) [75,78,79] which determines the substrate specificity of CusA and plays an important role in Ag(I) and Cu(I) transport directly from the cytoplasm [75,76]. Any mutation of that motif may result in decreased bacterial resistance to silver or copper ions [80]. When a substrate binds to the methionine motif, the conformation of CusA change, opening the way to the periplasmic part of CusC. It has to be said that there are more methionine residues in the structure of CusA. There are three pairs below (M410-M501, M403-M486, M391-M1009) and one pair (M271-M755) above the main binding site in the periplasmic space [75,76,78]. Those residues participate probably in the stepwise transport of heavy metal ions from the cytoplasm to the periplasmic part of CusC [76,78]. It is proposed that CusA has the ability to bind copper and silver ions from the periplasmic space. A similar function may be assigned to CusB, a membrane fusion protein (379 amino acids). Among MFP, CusB has a unique structure. It consists of two protomers with 3 β-domains (first domain interacts with periplasmic part of CusA) and 1 α-helical domain—responsible for the interaction with CusC. CusB serves as a protein span binding together CusA and CusC transporters [75,78]. The other role of CusB in Ag (I) and Cu (I) extrusion is strictly connected with CusF. It is a periplasmic chaperone with a structure of 5-stranded β-barrel consisting of three antiparallel 3-stranded β-sheets. It is produced only in the presence of heavy-metal ions. It binds silver and copper ions with two-methionine or two-cysteine sites and transports them to CusB, which triggers fast and reversible changes in conformation of CusA [75,79,80]. In the next step, CusF will be delivering Ag(I) or Cu(I) directly to CusA where ions will be extruded to CusC (457 amino acids). That outer membrane factor of CusCFBA, which is a cylindrical homotrimer formed of three subunits with a α/β-barrel structure, will funnel heavy-metal ions straight to the extracellular environment [75,79].

In 1975, *S.* Typhimurium caused the death of several patients from the burn ward of Massachusetts General Hospital. The isolated pathogen was silver resistant due to the presence of a 180 kb plasmid, known as pMG101 [81,82]. The pMG101 plasmid determines bacterial resistance to heavy metals (Hg, Ag), tellurite and a few antibiotics—chloramphenicol, streptomycin, tetracycline and ampicillin [43,83]. The specific region of pMG101 plasmid involved in resistance to silver, contains nine genes encoding Sil proteins which are gathered in three transcriptional units: *silRS*, *silE* and *silCFBAGP* (each controlled by a separate promoter) [43,84]. The function of SilG is still not known—the rest of the Sil proteins were characterized due to their homology to other resistance mechanisms, e.g., CusCFBA [43,81]. Some of the *sil* genes were identified in another bacteria genus, such as *Enterobacter*, *Klebsiella*, *Escherichia*, *Pseudomonas* or MRSA [84].

**Figure 5:**
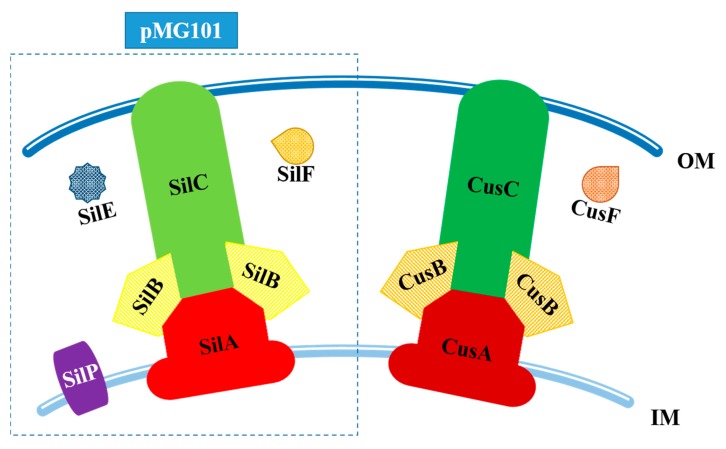
A comparison of the Sil (pMG101) and Cus silver resistance systems in Gram-negative bacteria, IM—inner membrane, OM—outer membrane.

The similarity in protein sequences between CusCFBA and SilCFBA reaches 80% [84]. In the exogenous mechanism of resistance to Ag(I), the tripartite RND efflux pumps are made from SilA, SilB and SilC [43]. The function of those proteins is homologous to the role of Cus system components. SilA exists as IM substrate-binding transporter, responsible for the uptake of silver ions from cytoplasm. SilB, as a membrane fusion protein, spans together each pump’s components and SilC, located in the OM, funnels captured ions to the extracellular environment [82,83]. The transcription of *sil* genes is carried out by a two-component regulatory system. It consists of the SilS protein—membrane histidine kinase and the response regulator (SilR). Those two components are homologs of products encoded by the *CusRS* operon [44,82]. In the silver resistance mechanism determined by Sil proteins, there are two periplasmic chaperones, SilF and SilE. SilE is an α-helix homolog of PcoE (sequence identity score: 48%), a protein that is able to bind copper ions from periplasmic space. Due to the presence of methionine and histidine residues in its structure, SilE is able to bind eight silver ions. SilF, a β-sheet homolog of CusF, is able to bind only single Ag^+^ [81,83]. The function of those two chaperones differs substantially: SilE is involved in the uptake of silver ions from periplasmic space, while SilF captures Ag(I) which got inside from extracellular space [43,81]. Both chaperones deliver their cargos to SilCBA. The last *sil* gene on pMG101 encodes SilP—inner membrane P-type ATP-ase. This protein transports silver ions from cytoplasm to the periplasmic space, where Ag^+^ will be bound by SilE [84]. A comparison of exogenous and endogenous mechanism of resistance to silver ions in Gram-negative bacteria is presented in Figure 5.

Apparently, the silver resistance does not widely occur in Gram-positive bacteria, specifically in staphylococci. Although MIC of Ag^+^ for *S. aureus* was estimated between 16 and 32 µg/mL [41,85], no resistance strains were selected during long-term (42 days) exposition to silver ions in 876 strains of *S. aureus* [85]. It is very interesting that antibiotic resistance occurs frequently and usually within a few days (e.g., with frequency 3 × 10^−6^ for fusacid) [85]. Therefore, silver ions remain a promising agent in prevention and treatment infection caused by *S. aureus*. Loh et al. [85] checked the prevalence of *sil* genes in 36 (33 MRSA strains and 3 MR-CNS (methycilin-resistant coagulase-negative *S. aureus*)) strains isolated from wounds and nasal sources in human and animals. They indicated *silE* only in 2 of 33 and 1 of 3 strains. This *silE* gene was a homologue in 95–100% to *silE* gene located on the pMG101 plasmid in Gram-negative bacteria. It is possible that the *silE* gene is not expressed in those cells [85]. It is interesting that the time of MRSA *silE-*positive death was 16 times longer than MRSA *silE*-negative. Sütterlin et al. [41] also proved that none of the tested *S. aureus* was *sil*-positive, neither developed resistance to silver after exposure to silver-based surgical dressings (duration of treatment: 2–14 months). 

## 5. Summary and Conclusions

We presented the similarities and differences in the mode of silver action on Gram-positive and Gram-negative bacteria. We noticed a gap in knowledge about the molecular mechanism of bacteria, both Gram-positive and Gram-negative, to silver nanoparticles. If we assume that silver nanoparticles are silver ions source, it is possible that the molecular mechanism of bacterial resistance is analogue to mechanism described for Ag^+^. If there is another way of antibacterial toxicity of silver nanoparticles, it is likely that different mechanisms of resistance to silver nanoparticle exist, for both Gram-negative and Gram-positive bacteria. The variety of silver nanoforms causes that every product with silver nanoparticles should be considered separately as a compound with different physico-chemical properties, different mode of action and different mechanisms of resistance.

## Figures and Tables

**Table 1:** Overview of certain nanocomposites of silver: their physico-chemical description and biological activity.

Nanocomposite (Named According to the Reference)	Silver Nanoparticles Size	Silver Nanoparticles Shape	Silver Amount in Nanocomposites	Form of Compound (If Applicable)	Type of Synthesis	Antibacterial Activity	References
Silver nanoparticles	10–15 nm	spherical, polyhedral	n/a	n/a	chemical	Antibacterial effect was dose-dependent. Tested silver nanoparticles were more effective against Gram-negative bacteria than Gram-positive; MoA: binding to the cell wall and penetrating it; modulation of cellular signalling	[18]
AgNPs	5–30 nm	variable: most spherical	n/a	n/a	biological	Increased antibacterial activity of antibiotics in the presence of AgNPs; MoA: binding to proteins (by thiol groups) and DNA	[19]
Silver nanoparticles	39 nm (spherical), 40 nm (triangular), 133–192 nm, diameter: 16 nm (rod-shaped)	variable: most spherical, triangular, rod-shaped	n/a	n/a	chemical	Inhibition of *Escherichia coli* growth on medium with silver nanoparticles; MoA: damage of bacterial cell membrane on multiple locations, formation of irregular pits	[20]
Nano-Ag	9.3 ± 2.8 nm	spherical	n/a	n/a	chemical	Inhibition of *E. coli* growth at 6 µM concentration of Nano-Ag; MoA: changes in expression of genes encoding envelope proteins (accumulation of envelope protein precursors), destabilization of outer membrane, disturbance of proton motive force	[21]
Silver nanoparticles	12 nm	spherical	n/a	n/a	chemical	Inhibition of *E. coli* growth at 50–60 µg/mL concentration of silver nanoparticles; MoA: damage of membranes, incorporation of silver nanoparticles into membranes, forming pits, disturbances in permeability	[22]
Silver nanoparticles	16 ± 8 nm, 21 ± 18 nm	icosahedral, twinned, decahedral	n/a	agglomerated inside the carbon matrix	chemical	Inhibition of Gram-negative and Gram-positive bacteria growth at 75 μg/mL concentration of silver nanoparticles; MoA: binding to cell membrane, permeability changes, disturbances in respiration process, penetration of the bacterial membranes, interacting with DNA, releasing silver ions	[23]
Ti/Ag	not specified	not specified	1.93–6.08% [m/m]	nanotexture, rutile, anatase	biological	Inhibition of *S. aureus* (MRSA) and *E. coli* growth at 15–75 µM; MoA: not specified	[24]
Nanosilver/diatomite	1–20 nm	spherical particles	0.537% [m/m]	not specified	chemical	0.5 g nanosilver/diatomite kills above 99% of *E. coli* within 30 min; MoA: not specified	[25]
Chitosan-AgNps	8–28 nm	spherical	1% [m/m]	chitosan/alginate nanofibers	chemical	Inhibition of *E. coli*, *Klebsiella pneumoniae*, *Bacillus pumilus* and *Staphylococcus aureus* growth; MoA: not specified	[26]
AgNps	15–160 nm (mean diameter 60 ± 10 nm)	spherical and irregular	n/a	not specified	biological	Inhibition of multidrug (MDR) pathogens: *Acinetobacter baumannii*, *E. coli*, *Pseudomonas aeruginosa* and *Salmonella enterica* growth at 25–50 µg concentration; MoA: not specified	[27]
GO-l-cys-AgNps	31.5–42 nm (mean diameter 35.34 ± 0.2 nm)	spherical	not specified	graphene sheets functionalized with l-cysteine	chemical	Inhibition of *E. coli* growth MoA: damages of the cell membrane	[28]
AgNPs	6–26 nm, 4.24–23.22 nm	spherical	n/a	foam	biological	Inhibition of the Gram-positive and Gram-negative bacteria growth at 676.9 mg/L concentration; MoA: not specified	[29]
Ag-NPs	100 nm, 30 nm diameter 200–300 length	Spherical, rod-like	n/a	oil microemulsion	chemical	Inhibition of *E. coli* and *S. aureus* growth at 0.05 mg/L; MoA: not specified	[30]
AgNPs	15 nm	spherical	n/a	n/a	biological	Inhibition of *E. coli*, *S. aureus*, *P. aeruginosa* growth at 50 µg/mL; MoA: not specified	[31]
AgNPs	5–40 nm	variable: spherical or rod-like	n/a	n/a	biological	Increased antibacterial activity of ampicillin, erythromycin and chloramphenicol in the presence of AgNPs (*E. coli*, *Salmonella* Typhi, *S. aureus*, *Micrococcus luteus*); MoA: not specified	[32]

n/a—not applicable; MoA—Mode of antibacterial action of silver; AgNPs—silver nanoparticles; GO—graphene oxide; MDR—multi-drug resistant; MRSA—Methicillin-resistant *S. aureus*.

## Abbreviations

ABC	ATP-binding cassette
ABNC	Active but nonculturable state
AgNPs	Silver nanoparticles
ATP	Adenosine triphosphate
CM	Cytoplasmic membrane
DNA	Deoxyribonucleic acid
GO	Graphene oxide
GSH	Glutathione
HME	Heavy-metal efflux
IM	Inner membrane
MATE	Multidrug and toxic compound extrusion
MBC	Minimal bactericidal concentration
MDR	Multidrug resistant
MFP	Periplasmic membrane fusion protein
MFS	Major facilitator superfamily
MIC	Minimal inhibitory concentration
MoA	Mode of antibacterial action of silver
MR-CNS	Methycilin-resistant coagulase-negative *S. aureus*
MRSA	Methicillin-resistant *S. aureus*
n/a	Not applicable
OM	Outer membrane
OMF	Outer membrane factor
OMP	Outer membrane protein
O_2_^−^•	Superoxide anion
OH•	Hydroxyl radical
PEG	Polyethylene glycol
RND	Resistance-nodulation-division
ROS	Reactive oxygen species
SMR	Small multidrug resistance
TEM	Transmission electron microscopy

## References

[1] Klasen H.J. (2000). Historical review of the use of silver in the treatment of burns. I. Early uses. Burn. J. Int. Soc. Burn Inj..

[2] Klasen H.J. (2000). A historical review of the use of silver in the treatment of burns. II. Renewed interest for silver. Burns.

[3] Benli B., Yalın C. (2017). The influence of silver and copper ions on the antibacterial activity and local electrical properties of single sepiolite fiber: A conductive atomic force microscopy (C-AFM) study. Appl. Clay Sci..

[4] Sun Z., Fan C., Tang X., Zhao J., Song Y., Shao Z., Xu L. (2016). Characterization and antibacterial properties of porous fibers containing silver ions. Appl. Surf. Sci..

[5] Chen R., Ni H., Zhang H., Yue G., Zhan W., Xiong P. (2013). A preliminary study on antibacterial mechanisms of silver ions implanted stainless steel. Vacuum.

[6] Mohiti-Asli M., Pourdeyhimi B., Loboa E.G. (2014). Novel, silver-ion-releasing nanofibrous scaffolds exhibit excellent antibacterial efficacy without the use of silver nanoparticles. Acta Biomater..

[7] Dong H., Gao Y., Sinko P.J., Wu Z., Xu J., Jia L. (2016). The nanotechnology race between China and the United States. Nano Today.

[8] Ying J.Y. (2008). The era of nanotechnology. Nano Today.

[9] Sheng Z., Liu Y. (2017). Potential impacts of silver nanoparticles on bacteria in the aquatic environment. J. Environ. Manag..

[10] Shang L., Dong S., Nienhaus G.U. (2011). Ultra-small fluorescent metal nanoclusters: Synthesis and biological applications. Nano Today.

[11] Zheng K., Yuan X., Goswami N., Zhang Q., Xie J. (2014). Recent advances in the synthesis, characterization and biomedical applications of ultrasmall thiolated silver nanoclusters. RSC Adv..

[12] Banhart F., Kotakoski J., Krasheninnikov A.V. (2011). Structural Defects in Graphene. ACS Nano.

[13] Kharissova O.V., Dias H.V.R., Kharisov B.I., Pérez B.O., Pérez V.M.J. (2013). The greener synthesis of nanoparticles. Trends Biotechnol..

[14] Singh P., Kim Y.-J., Zhang D., Yang D.-C. (2016). Biological Synthesis of Nanoparticles from Plants and Microorganisms. Trends Biotechnol..

[15] Mittal A.K., Chisti Y., Banerjee U.C. (2013). Synthesis of metallic nanoparticles using plant extracts. Biotechnol. Adv..

[16] Sharma V.K., Yngard R.A., Lin Y. (2009). Silver nanoparticles: Green synthesis and their antimicrobial activities. Adv. Colloid Interface Sci..

[17] Jyoti K., Baunthiyal M., Singh A. (2016). Characterization of silver nanoparticles synthesized using *Urtica dioica* Linn. leaves and their synergistic effects with antibiotics. J. Radiat. Res. Appl. Sci..

[18] Shrivastava S., Bera T., Roy A., Singh G., Ramachandrarao P., Dash D. (2007). Characterization of enhanced antibacterial effects of novel silver nanoparticles. Nanotechnology.

[19] Naqvi S.Z.H., Kiran U., Ali M.I., Jamal A., Hameed A., Ahmed S., Ali N. (2013). Combined efficacy of biologically synthesized silver nanoparticles and different antibiotics against multidrug-resistant bacteria. Int. J. Nanomed..

[20] Pal S., Tak Y.K., Song J.M. (2007). Does the antibacterial activity of silver nanoparticles depend on the shape of the nanoparticle? A study of the Gram-negative bacterium *Escherichia coli*. Appl. Environ. Microbiol..

[21] Lok C.-N., Ho C.-M., Chen R., He Q.-Y., Yu W.-Y., Sun H., Tam P.K.-H., Chiu J.-F., Che C.-M. (2006). Proteomic analysis of the mode of antibacterial action of silver nanoparticles. J. Proteome Res..

[22] Sondi I., Salopek-Sondi B. (2004). Silver nanoparticles as antimicrobial agent: A case study on *E. coli* as a model for Gram-negative bacteria. J. Colloid Interface Sci..

[23] Morones J.R., Elechiguerra J.L., Camacho A., Holt K., Kouri J.B., Ramírez J.T., Yacaman M.J. (2005). The bactericidal effect of silver nanoparticles. Nanotechnology.

[24] Mohandas A., Krishnan A.G., Biswas R., Menon D., Nair M.B. (2017). Antibacterial and cytocompatible nanotextured Ti surface incorporating silver via single step hydrothermal processing. Mater. Sci. Eng. C Mater. Biol. Appl..

[25] Xia Y., Jiang X., Zhang J., Lin M., Tang X., Zhang J., Liu H. (2017). Synthesis and characterization of antimicrobial nanosilver/diatomite nanocomposites and its water treatment application. Appl. Surf. Sci..

[26] Mokhena T.C., Luyt A.S. (2017). Electrospun alginate nanofibres impregnated with silver nanoparticles: Preparation, morphology and antibacterial properties. Carbohydr. Polym..

[27] Raman G., Park S.J., Sakthivel N., Suresh A.K. (2017). Physico-cultural parameters during AgNPs biotransformation with bactericidal activity against human pathogens. Enzym. Microb. Technol..

[28] Chandraker K., Nagwanshi R., Jadhav S.K., Ghosh K.K., Satnami M.L. (2017). Antibacterial properties of amino acid functionalized silver nanoparticles decorated on graphene oxide sheets. Spectrochim. Acta Part A Mol. Biomol. Spectrosc..

[29] Moustafa M.T. (2017). Removal of pathogenic bacteria from wastewater using silver nanoparticles synthesized by two fungal species. Water Sci..

[30] Gao H., Yang H., Wang C. (2017). Controllable preparation and mechanism of nano-silver mediated by the microemulsion system of the clove oil. Results Phys..

[31] Bindhu M.R., Umadevi M. (2015). Antibacterial and catalytic activities of green synthesized silver nanoparticles. Spectrochim. Acta Part A Mol. Biomol. Spectrosc..

[32] Fayaz A.M., Balaji K., Girilal M., Yadav R., Kalaichelvan P.T., Venketesan R. (2010). Biogenic synthesis of silver nanoparticles and their synergistic effect with antibiotics: A study against gram-positive and gram-negative bacteria. Nanomed. Nanotechnol. Biol. Med..

[33] Bruins M.R., Kapil S., Oehme F.W. (2000). Microbial resistance to metals in the environment. Ecotoxicol. Environ. Saf..

[34] Roebben G., Rauscher H., Amenta V., Aschberger K., Boix Sanfeliu A., Calzolai L., Emons H., Gaillard C., Gibson N., Holzwarth U. (2014). Recommendation of European Union 2011/696/UE.

[35] Parveen R., Shamsi T.N., Fatima S. (2017). Nanoparticles-protein interaction: Role in protein aggregation and clinical implications. Int. J. Biol. Macromol..

[36] Li L., Zhao C., Zhang Y., Yao J., Yang W., Hu Q., Wang C., Cao C. (2017). Effect of stable antimicrobial nano-silver packaging on inhibiting mildew and in storage of rice. Food Chem..

[37] Jinu U., Gomathi M., Saiqa I., Geetha N., Benelli G., Venkatachalam P. (2017). Green engineered biomolecule-capped silver and copper nanohybrids using *Prosopis cineraria* leaf extract: Enhanced antibacterial activity against microbial pathogens of public health relevance and cytotoxicity on human breast cancer cells (MCF-7). Microb. Pathog..

[38] Ballottin D., Fulaz S., Cabrini F., Tsukamoto J., Durán N., Alves O.L., Tasic L. (2017). Antimicrobial textiles: Biogenic silver nanoparticles against *Candida* and *Xanthomonas*. Mater. Sci. Eng. C.

[39] Li W.-R., Sun T.-L., Zhou S.-L., Ma Y.-K., Shi Q.-S., Xie X.-B., Huang X.-M. (2017). A comparative analysis of antibacterial activity, dynamics and effects of silver ions and silver nanoparticles against four bacterial strains. Int. Biodeterior. Biodegrad..

[40] Jung W.K., Koo H.C., Kim K.W., Shin S., Kim S.H., Park Y.H. (2008). Antibacterial Activity and Mechanism of Action of the Silver Ion in *Staphylococcus aureus* and *Escherichia coli*. Appl. Environ. Microbiol..

[41] Sütterlin S., Tano E., Bergsten A., Tallberg A.-B., Melhus A. (2012). Effects of silver-based wound dressings on the bacterial flora in chronic leg ulcers and its susceptibility in vitro to silver. Acta Derm. Venereol..

[42] Percival S.L., Bowler P.G., Russell D. (2005). Bacterial resistance to silver in wound care. J. Hosp. Infect..

[43] Randall C.P., Gupta A., Jackson N., Busse D., O’Neill A.J. (2015). Silver resistance in Gram-negative bacteria: A dissection of endogenous and exogenous mechanisms. J. Antimicrob. Chemother..

[44] Randall C.P., Oyama L.B., Bostock J.M., Chopra I., O’Neill A.J. (2013). The silver cation (Ag^+^): Antistaphylococcal activity, mode of action and resistance studies. J. Antimicrob. Chemother..

[45] Yamanaka M., Hara K., Kudo J. (2005). Bactericidal Actions of a Silver Ion Solution on *Escherichia coli*, Studied by Energy-Filtering Transmission Electron Microscopy and Proteomic Analysis. Appl. Environ. Microbiol..

[46] Koebnik R., Locher K.P., Van Gelder P. (2000). Structure and function of bacterial outer membrane proteins: Barrels in a nutshell. Mol. Microbiol..

[47] Schulz G.E. (2002). The structure of bacterial outer membrane proteins. Biochim. Biophys. Acta.

[48] Radzig M.A., Nadtochenko V.A., Koksharova O.A., Kiwi J., Lipasova V.A., Khmel I.A. (2013). Antibacterial effects of silver nanoparticles on gram-negative bacteria: Influence on the growth and biofilms formation, mechanisms of action. Colloids Surf. B Biointerfaces.

[49] Yen M.R., Peabody C.R., Partovi S.M., Zhai Y., Tseng Y.H., Saier M.H. (2002). Protein-translocating outer membrane porins of Gram-negative bacteria. Biochim. Biophys. Acta.

[50] Li X.Z., Nikaido H., Williams K.E. (1997). Silver-resistant mutants of *Escherichia coli* display active efflux of Ag^+^ and are deficient in porins. J. Bacteriol..

[51] Feng Q.L., Wu J., Chen G.Q., Cui F.Z., Kim T.N., Kim J.O. (2000). A mechanistic study of the antibacterial effect of silver ions on *Escherichia coli* and *Staphylococcus aureus*. J. Biomed. Mater. Res..

[52] Dakal T.C., Kumar A., Majumdar R.S., Yadav V. (2016). Mechanistic Basis of Antimicrobial Actions of Silver Nanoparticles. Front. Microbiol..

[53] Xiu Z.-M., Ma J., Alvarez P.J.J. (2011). Differential effect of common ligands and molecular oxygen on antimicrobial activity of silver nanoparticles versus silver ions. Environ. Sci. Technol..

[54] Lok C.-N., Ho C.-M., Chen R., He Q.-Y., Yu W.-Y., Sun H., Tam P.K.-H., Chiu J.-F., Che C.-M. (2007). Silver nanoparticles: Partial oxidation and antibacterial activities. J. Biol. Inorg. Chem..

[55] Prabhu S., Poulose E.K. (2012). Silver nanoparticles: Mechanism of antimicrobial action, synthesis, medical applications and toxicity effects. Int. Nano Lett..

[56] Rai M.K., Deshmukh S.D., Ingle A.P., Gade A.K. (2012). Silver nanoparticles: The powerful nanoweapon against multidrug-resistant bacteria. J. Appl. Microbiol..

[57] Dubey P., Matai I., Kumar S.U., Sachdev A., Bhushan B., Gopinath P. (2015). Perturbation of cellular mechanistic system by silver nanoparticle toxicity: Cytotoxic, genotoxic and epigenetic potentials. Adv. Colloid Interface Sci..

[58] Ramalingam B., Parandhaman T., Das S.K. (2016). Antibacterial Effects of Biosynthesized Silver Nanoparticles on Surface Ultrastructure and Nanomechanical Properties of Gram-Negative Bacteria viz. *Escherichia coli* and *Pseudomonas aeruginosa*. ACS Appl. Mater. Interfaces.

[59] Le Pape H., Solano-Serena F., Contini P., Devillers C., Maftah A., Leprat P. (2004). Involvement of reactive oxygen species in the bactericidal activity of activated carbon fibre supporting silver; Bactericidal activity of ACF(Ag) mediated by ROS. J. Inorg. Biochem..

[60] Joshi N., Ngwenya B.T., Butler I.B., French C.E. (2015). Use of bioreporters and deletion mutants reveals ionic silver and ROS to be equally important in silver nanotoxicity. J. Hazard. Mater..

[61] Park H.-J., Kim J.Y., Kim J., Lee J.-H., Hahn J.-S., Gu M.B., Yoon J. (2009). Silver-ion-mediated reactive oxygen species generation affecting bactericidal activity. Water Res..

[62] Li M., Yin J.-J., Wamer W.G., Lo Y.M. (2014). Mechanistic characterization of titanium dioxide nanoparticle-induced toxicity using electron spin resonance. J. Food Drug Anal..

[63] Choi O., Hu Z. (2008). Size dependent and reactive oxygen species related nanosilver toxicity to nitrifying bacteria. Environ. Sci. Technol..

[64] Mandal D., Kumar Dash S., Das B., Chattopadhyay S., Ghosh T., Das D., Roy S. (2016). Bio-fabricated silver nanoparticles preferentially targets Gram positive depending on cell surface charge. Biomed. Pharmacother..

[65] Gitipour A., Al-Abed S.R., Thiel S.W., Scheckel K.G., Tolaymat T. (2017). Nanosilver as a disinfectant in dental unit waterlines: Assessment of the physicochemical transformations of the AgNPs. Chemosphere.

[66] McGillicuddy E., Murray I., Kavanagh S., Morrison L., Fogarty A., Cormican M., Dockery P., Prendergast M., Rowan N., Morris D. (2017). Silver nanoparticles in the environment: Sources, detection and ecotoxicology. Sci. Total Environ..

[67] Xiu Z., Zhang Q., Puppala H.L., Colvin V.L., Alvarez P.J.J. (2012). Negligible particle-specific antibacterial activity of silver nanoparticles. Nano Lett..

[68] Sotiriou G.A., Meyer A., Knijnenburg J.T.N., Panke S., Pratsinis S.E. (2012). Quantifying the Origin of Released Ag^+^ Ions from Nanosilver. Langmuir.

[69] Durán N., Marcato P.D., Conti R.D., Alves O.L., Costa F.T.M., Brocchi M. (2010). Potential use of silver nanoparticles on pathogenic bacteria, their toxicity and possible mechanisms of action. J. Braz. Chem. Soc..

[70] Izak-Nau E., Huk A., Reidy B., Uggerud H., Vadset M., Eiden S., Voetz M., Himly M., Duschl A., Dusinska M. (2015). Impact of storage conditions and storage time on silver nanoparticles’ physicochemical properties and implications for their biological effects. RSC Adv..

[71] Mathema V.B., Thakuri B.C., Sillanpää M. (2011). Bacterial *mer* operon-mediated detoxification of mercurial compounds: A short review. Arch. Microbiol..

[72] Martinez J.L., Sánchez M.B., Martínez-Solano L., Hernandez A., Garmendia L., Fajardo A., Alvarez-Ortega C. (2009). Functional role of bacterial multidrug efflux pumps in microbial natural ecosystems. FEMS Microbiol. Rev..

[73] Piddock L.J.V. (2006). Multidrug-resistance efflux pumps—Not just for resistance. Nat. Rev. Microbiol..

[74] Fernando D.M., Kumar A. (2013). Resistance-Nodulation-Division Multidrug Efflux Pumps in Gram-Negative Bacteria: Role in Virulence. Antibiotics.

[75] Delmar J.A., Su C.-C., Yu E.W. (2014). Bacterial multidrug efflux transporters. Annu. Rev. Biophys..

[76] Long F., Su C.-C., Zimmermann M.T., Boyken S.E., Rajashankar K.R., Jernigan R.L., Yu E.W. (2010). Crystal structures of the CusA efflux pump suggest methionine-mediated metal transport. Nature.

[77] Su C.-C., Long F., Lei H.-T., Bolla J.R., Do S.V., Rajashankar K.R., Yu E.W. (2012). Charged amino acids (R83, E567, D617, E625, R669 and K678) of CusA are required for metal ion transport in the Cus efflux system. J. Mol. Biol..

[78] Su C.-C., Long F., Yu E.W. (2011). The Cus efflux system removes toxic ions via a methionine shuttle. Protein Sci..

[79] Chacón K.N., Mealman T.D., McEvoy M.M., Blackburn N.J. (2014). Tracking metal ions through a Cu/Ag efflux pump assigns the functional roles of the periplasmic proteins. Proc. Natl. Acad. Sci. USA.

[80] Franke S., Grass G., Rensing C., Nies D.H. (2003). Molecular analysis of the copper-transporting efflux system CusCFBA of *Escherichia coli*. J. Bacteriol..

[81] Asiani K.R., Williams H., Bird L., Jenner M., Searle M.S., Hobman J.L., Scott D.J., Soultanas P. (2016). SilE is an intrinsically disordered periplasmic “molecular sponge” involved in bacterial silver resistance. Mol. Microbiol..

[82] Silver S. (2003). Bacterial silver resistance: Molecular biology and uses and misuses of silver compounds. FEMS Microbiol. Rev..

[83] Silver S., Gupta A., Matsui K., Lo J.F. (1999). Resistance to Ag(I) cations in bacteria: Environments, genes and proteins. Met.-Based Drugs.

[84] Finley P.J., Norton R., Austin C., Mitchell A., Zank S., Durham P. (2015). Unprecedented Silver Resistance in Clinically Isolated Enterobacteriaceae: Major Implications for Burn and Wound Management. Antimicrob. Agents Chemother..

[85] Loh J.V., Percival S.L., Woods E.J., Williams N.J., Cochrane C.A. (2009). Silver resistance in MRSA isolated from wound and nasal sources in humans and animals. Int. Wound J..

